# Child Life under Queen Victoria

**Published:** 1897-03-06

**Authors:** 


### Child Life under Queen Victoria.

### IV.—The Colliery Children.

It was necessary to deal in some detail with the children in textile factories because it may truly be said of them that they fought the battle for all the others. Action on behalf of the children—first the pauper apprentices and then the "free" workers—in the cotton factories, established the principle that children, as not being free agents in the sale of their labour, had a right to be protected from the cupidity of both employers and parents, and, as being the men and women of the coming generation, had a national right to such conditions as were necessary to their healthy development. Even before their battle was fought out, these principles were so applied to other cases.

For generations the coal miners had been held to be a degraded race. They had been serfs, to be hired out or sold with the mines they worked in, and even after that outrage to the name of freedom had been swept away, the degradation of slavery remained, and the name of collier was a synonym for ignorance and brutality. Anyone who had inquired into the conditions of a collier's life sixty years ago would have found abundant explanation of every fault in his nature. The children were ignorant, for they were sent down the pit when they were little more than babies; the parents were brutal, because the sin of such treatment of their children had never been brought before them.

The present writer is acquainted with a man who went "down the pit" when he was seven years old. Between seven and nine was the average age for children—boys and girls alike—to begin work, but some infants were sent down at four or five. They could not then do any very hard work, but they were fit to be "trappers." Improvements in the ventilation of mines have made it possible to do without "trappers"; and it is necessary to explain what their functions were. The passages in the mine were not all open, but were shut off in sections by traps or doors. This served to concentrate the cool air from the shaft in the parts where work was going on; whereas, without them, the heat might have become unendurable, or gases, which would have caused death by explosion or otherwise, might have affected the miners. But since the trucks on which the coals were taken to the shaft had often to pass through these shut-off passages, a little child, the trapper, was set at each door, to open it when he heard the "whirley," or truck approaching, and shut it after it had passed. It is obvious that anyone, however young, could fulfil an office so mechanical; while it is equally obvious that the mechanical and monotonous nature of the work made it peculiarly deadening to the faculties of children. They sat behind their traps in utter darkness for twelve or fourteen hours, with no more interesting task than pulling the door open and shutting it again. If they strayed for more than a few yards from their posts they might delay the passage of a truck, in which case they would be savagely beaten. The ground they sat on was damp, and many mines were infested with vermin. Stories are told of rats grown so bold that they would steal the miners' food in their presence, and of an occasional one who ran off with a lighted candle in its mouth, and consequently exploded the gas in a "fiery" part of the mine. In such circumstances terrors of the imagination must have added not a little to the little worker's misery.

This was, however, the easiest part of the young collier's life. Boy or girl—the sex mattered not—was soon put to "hurrying," an expressive term for pushing the little trucks of coal along the passages to the mouth of the shaft. Women also worked at this, but we do not propose here to speak of the collier women, their hardships, their degradation, or the injuries entailing lifelong suffering which their work made common amongst them. For low, narrow passages—some were not more than eighteen to twenty-four inches in height—children were preferred. Sometimes they crawled along on their knees, pushing the trucks before them. Another method was to put a belt round the "hurryer's" waist, and fasten to this a chain that passed between the legs and was hooked to the coal truck. Then, sometimes crouching double, sometimes crawling on hands and knees, they dragged their burdens after them.

In those days there was no cage at the shaft, raised and lowered by machinery, to take up the coals. Women, girls, and children carried the creels, or baskets, of coal up a risky winding stair, sometimes rotten, often unrailed, from the bottom of the mine to the pithead. The baskets were piled high, and there was every chance that if any pieces fell off one basket they would injure the bearer of the succeeding one. Fatal accidents occasionally occurred thus. Even without such risks it seems pitiful enough to think of children of seven years of age carrying "burdens of coal of half a hundredweight up steps that, in the aggregate, equalled an ascent 14 times a day to the summit of St. Paul's Cathedral." Another task which was often assigned to children was pumping at the pit bottom. And at all these tasks they laboured as long as adults. Twelve or 14 hours was the average, but sometimes in times of pressure they had to work "double shifts"—36 hours at a stretch!

Much of this work is now done by machinery; and, indeed, there is no better way to arouse the inventive mind of man than to deprive him of cheap human labour. The remainder is assigned to ponies, while, for the men and lads who still work in the pits, better machinery, better ventilations, and better regulations have greatly lessened the horrors of what is, at the best, a dreary mode of making a living. But if it had not been for the Commission demanded by Lord Ashley in 1840, and the report issued by it in 1842, the former hardships, and the cruelties which accompanied them under the hands of brutal overseers, might have gone on for many a year. The plea urged in extenuation of the continuance of this state of barbarism—it was nothing less!—in the midst of a Christian and civilised nation, was the old one, that without cheap child-labour the pits could not be worked at a profit, with the ingenious addition that "after a certain age the vertebræ of the back do not conform to the required positions, and therefore the children must begin early."

Fortunately, the horror felt by the whole nation at the revelations made by the Committee was so intense that the Bill brought forward by Lord Ashley to exclude women and children from work in mines passed with less than the usual opposition, and another class of children were restored to the privileges of childhood.

